# A new method to identify key match-play behaviours of young soccer players: Development of the Hull Soccer Behavioural Scoring Tool

**DOI:** 10.1371/journal.pone.0295953

**Published:** 2024-03-08

**Authors:** James Robinson, Sean Cumming, Jamie Salter, John Toner, Chris Towlson

**Affiliations:** 1 University of Hull, School of Sport, Exercise and Rehabilitation Sciences, Hull, United Kingdom; 2 Department for Health, University of Bath, Bath, United Kingdom; 3 York St John University, School of Science, Technology and Health, York, United Kingdom; Mugla Sitki Kocman University: Mugla Sitki Kocman Universitesi, TURKEY

## Abstract

The aim of this research was to assess the validity and reliability of a newly developed scoring tool, designed for monitoring youth soccer players during match-play performance to support coaches/scouts with the talent identification process. The method used to design the Hull Soccer Behavioural Scoring Tool comprised of a five-stage process of (i) conducting an initial literature review to establish content validity (ii) gaining content validity through a cross sectional online survey (iii) establishing face validity via expert coach feedback (iv) conducting inter-rater reliability tests and (v) intra-rater reliability tests. In stage two, twenty-two soccer academy practitioners completed an online survey, which revealed that player behaviours such as resilience, competitiveness, and decision making were all valued as the most important behavioural characteristics by practitioners (90.9%), whilst X-factor was valued as least important by a significant amount (27.2%). Stages three to five of the testing procedure included a sample of four academy coaches not involved in the preceding stage. Twenty male collegiate soccer players (under-16 to under-18) involved in the study took part in four versus four small-sided games (SSG) in a ‘round-robin’ tournament across three weeks which accumulated 14 SSG’s, 100–140 minutes of playing time and 70–98 individual player grades. Two of the four academy coaches watched the SSG’s and used the Hull Soccer Behavioural Scoring Tool to assess live evidence of desirable player behaviours, which was subsequently followed by retrospective video analysis for intra-rater reliability testing. The remaining two academy coaches watched the same SSG retrospective video footage to test for inter-rater reliability. Reliability results revealed an acceptable level of agreement with scores between 81.25%—89.9% for inter-rater whilst intra-rater provided scores between 80.35%—99.4%. Preliminary evidence here suggests that the Hull Soccer Behavioural Scoring Tool is both a valid and reliable method to assess desirable player behaviours during talent identification processes. Thus, youth soccer practitioners and researchers should seek to test and further validate the tool in order to confirm its utility as a means of measuring behavioural characteristics of youth soccer players.

## Introduction

In 2012, the English Premier League initiated the Elite Player Performance Plan (EPPP) as they sought to utilize different methods and improve talent development and identification, with a view to increasing the number of homegrown players [1]. Using the Football Associations (FA) Four Corner Model [2] as a template, the EPPP requires club practitioners to use the Performance Management Application (PMA) to subjectively and objectively evaluate players technical, tactical, physical, and psycho-social attributes to meet audit requirements [3,4]. Of these aforementioned attributes, physical performance can be measured objectively through anthropometry and various fitness tests, whereas the measurement of technical, tactical, and psycho-social attributes all rely on practitioners’ subjective judgements [4]. Whilst subjective beliefs can be important, they can also be problematic, given scouts and coaches use ‘instinct’, ‘gut feeling’ and take easily observable attributes into consideration when attempting to identify talent [5–7]. Thus, demonstrating the absence of an objective measure underpinning the talent identification system. Despite the obvious importance of being technically and tactically gifted, Towlson and colleagues [8] found soccer academy recruitment staff place greater value on psychological characteristics over technical/tactical and physical factors during talent selection, with attributes such as confidence, competitiveness, and positive attitude [9] appearing to be of highest importance.

The accurate assessment of desirable soccer-specific characteristics, however, is often confounded by the timing and tempo of biological maturity [10], which can influence the physical and psychological development of children [11,12] and selection of players for professional soccer academies [13,14]. This is evidenced by the psychological advantage (e.g., enhanced self-efficacy) late maturing player can possess over their early-maturing counterparts [15] across the development pathway. These psychological aspects are important for talent identification, as late-maturing players have been characterised as being achievement-oriented and highly skilled (between 13 and 14 years; [16]), which might be linked, in part, to the onset and cessation of peak height velocity (PHV) [17,18]. For instance, our previous findings have shown that performing in maturity mis-matched (i.e., late maturing versus early maturing) categorised groups of players (using ‘bio-banding’; [19–21]) during small-sided games provides late-maturing players with a mis-matched environment which allows them to exhibit a number of desirable psychological characteristics [22]. This maturity-related performance phenomena can be in part explained by the ‘underdog hypothesis’ [15,23], which proposes that late-maturing players may have established enhanced psychological skills that permit them to compete with their more mature team mates on equal terms [23]. This hypothesis is further underpinned by the suggestion that late maturing players have advanced self-regulatory skills, which characterises the degree to which individuals are metacognitively, motivationally, and behaviourally proactive participants in the learning process [24]. This is of importance and significance to talent identification practitioners as self-regulatory skills have been identified to distinguish elite athletes from their less-skilled counterparts [25]. However, despite the significance attributed to the development of psychological behaviours, there is no validated and reliable soccer specific behaviour scoring tool that can be used by coaches within soccer talent identification systems. Therefore, the aim of the study was to create a tool suitable to assist soccer clubs with holistic assessment for the ongoing (de)selection process.

## Methods

Following institutional ethical approval (University of Hull; REF FHS350), this study used a five-stage process which was informed by the work of Brewer and Jones [26] and Cushion and colleagues [27]. The process included: establishing content validity by conducting an initial literature review (stage one); establishing content validity with insight from industry practitioners (stage two); establishing face validity (stage three); and conducting inter-rater reliability (stage four) and intra-rater reliability (stage five).

### Establishing content validity of the Hull Soccer Behavioural Scoring Tool

#### Stage one—Literature review

**Fig 1:**
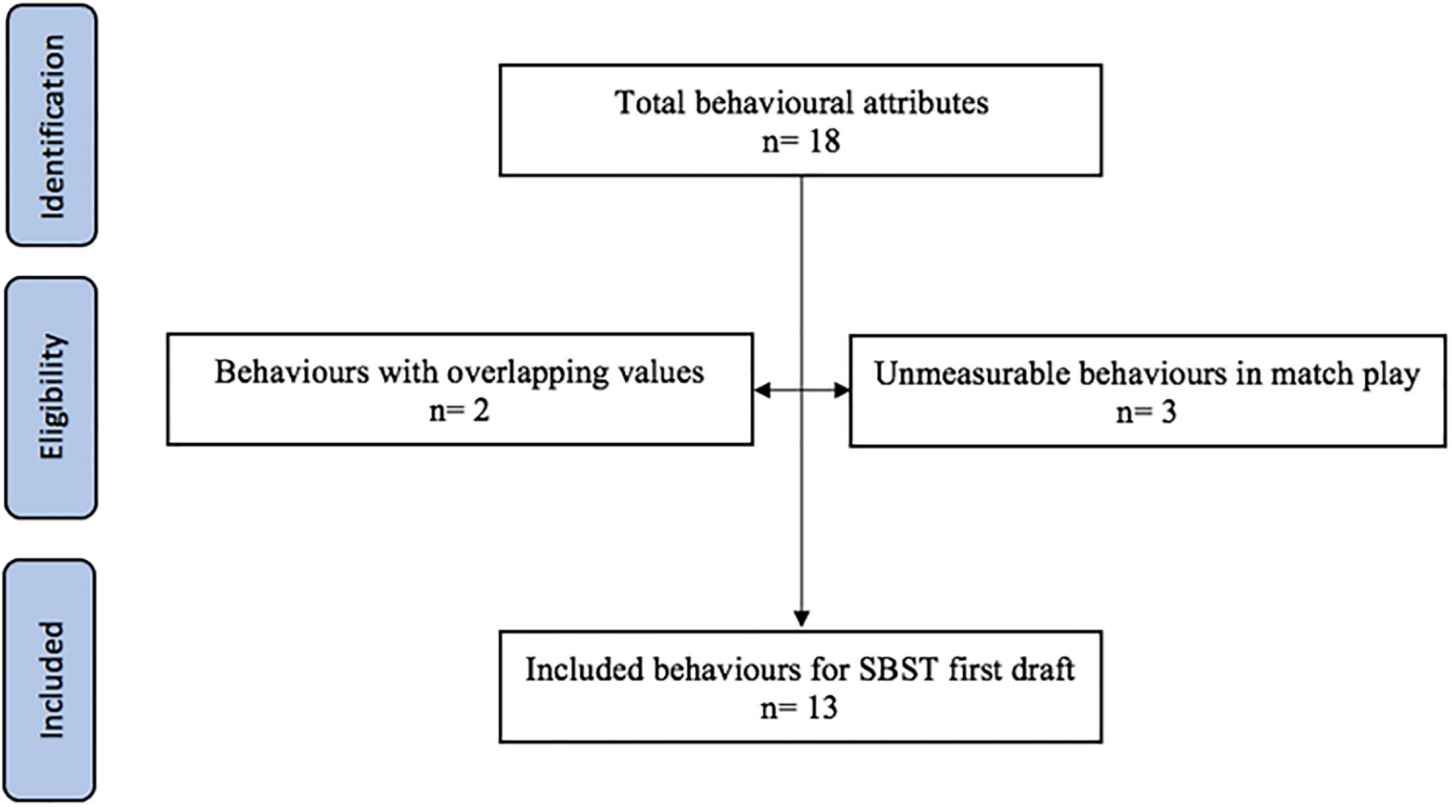
Flow chart of behavioural attribute selection and exclusion for the Hull Soccer Behavioural Scoring Tool.

Following previously outlined methods by Cushion and colleagues [27], the first step towards establishing content validity was to conduct a review of relevant literature on behavioural categories identified as being valuable for soccer player development and talent identification. Completion of a thorough review of the literature ensured all important aspects of psychological behaviours were examined [28] which confirmed thorough content validity was achieved [29]. Fig 1 details the process of the literature review, specifically outlining the included/excluded behaviours to be explored within the later stages of the study. The total number of behavioural attributes were characterised into measurable (e.g., could be seen in match play performance) or unmeasurable (e.g., could not be seen in match play performance) performance variables. In addition, following discussion within the research team who consisted of experienced academic researchers within talent identification and sports coaching, the behaviour ‘self-discipline’ was merged with ‘resilience’. Whilst ‘personality/character’ were merged with ‘competitiveness’ because the operational definitions observed for these behaviours were considered to be too similar in nature and had overlapping values.

The behavioural responses included ‘coachability’ [6,9,30], ‘adaptability’ [9], ‘decision making’ [9,31,32], ‘positive attitude’ [8,9,22,30,31], ‘resilience’ [8,33], ‘X-factor’ [8,9,22,32], ‘competitiveness’ [9,22,30], ‘confidence’ [8,9,22,34,35], ‘maintaining composure under pressure’ [8], ‘match presence’ [9,32,34], ‘communication’ [9,35], ‘on pitch bravery’ [8] and ‘anticipation’ [31,32,36].

#### Stage two—Establishing content validity

Using a previously published survey design [8,37] and in collaboration with the academic research team conducting the literature review, experienced coaches were contacted (via advertisements posted on Twitter, LinkedIn and through email circulation) and requested that they independently completed an online survey [38] to identify which behaviours practitioners considered as being important to evaluate during talent identification. This consisted of 67 questions (multiple choice: n = 8; 5-point Likert scale: n = 59) and took approximately 20 minutes to complete. Survey response data was collected between January (2022), with subsequent data analysis occurring in February (2022). Together, the three operational definitions per psychological behaviour, in conjunction with the survey allowed the research team to assess practitioners understanding of the definitions, whilst further providing a valid opinion of which definitions were to be used within the Hull Soccer Behavioural Scoring Tool before pilot testing.

Twenty-nine practitioners started the online survey [38]. Of these, 22 (75.9%) satisfied the inclusion/exclusion criteria (*Are you above the age of 18*?: n = 29 [100%]; *Have you worked at a professional soccer academy or development programme*?: n = 24 [82.8%]; *Do you consent to participate in this survey*?: n = 22 [91.6%]) and were included in the study. Section two of the survey required respondents to provide demographic details such as age (31.0 ± 22.0) and coaching experience (6.0 ± 8.0). The respondent was also required to state the phase of the EPPP they primarily coach within (i.e., Foundation Phase U9-11: n = 7 [31.8%]; Youth Phase U12-16: n = 7 [31.8%]; Professional Phase U18-23: n = 2 [9.1%]; First Team: n = 6 [27.3%]). Respondents further detailed their formal soccer qualifications (*Which coaching qualification do you hold*? None: n = 3 [13.6%]; FA level 1 coaching: n = 5 [22.7%]; FA level 2 coaching: n = 7 [31.8%]; FA UEFA B coaching licence: n = 5 [22.7%]; FA UEFA A coaching licence: n = 1 [4.5%]; FA UEFA Pro coaching licence: n = 1 [4.5%]; other commented responses were: FA Youth Award: n = 3 [13.6%]; On-going FA UEFA B coaching licence: n = 1 [4.5%]; Accredited Strength and Conditioning: n = 1 [4.5%]; Growth and Maturation: n = 1 [4.5%]) and relevant academic qualifications (*What is the highest academic qualification you hold in a relevant subject area*? None: n = 2 [9.1%]; Bachelor’s Degree: n = 9 [40.9%]; Master’s Degree: n = 5 [22.7%]; PhD/Doctoral Degree n = 4 [18.2%]; Higher National Diploma n = 0 [0%]; Other n = 2 [9.1%]).

Using the shortlisted variables identified within stage one, respondents were asked how important they consider each psychological behaviour metric is for talent identification, using a 5-point Likert scale (1—*least important*; 5—*most important*). Scores were then pooled (i.e., the sum of 1 and 2; 4 and 5) to rank the attributes in terms of their perceived importance. The selected operational definitions had to meet a ‘necessary’ criterion of >70% or were otherwise removed (On pitch bravery; Match presence) [39]. Respondents were then asked to identify which of the accompanying three to five operational definitions best represented each of the psychological behaviour metrics using a 5-point Likert scale (1 –*strongly disagree*; 5 –*strongly agree*). The total number of *strongly agree* and *agree*, accompanied by the total number of *strongly disagree* and *disagree* were both aggregated to identify which operational definition best represented the given psychological behaviour.

Two coaches (Coach 1: FA UEFA B coaching licence, 10 years professional coaching, PhD sport coaching and performance science; Coach 2: FA level 2, 4 years assistant manager at semi-professional level, PhD sport coaching and performance science) were given the opportunity to critically comment on the instruments useability and content, whilst also providing opinions on attributes they believed were either ambiguous, too difficult to objectify (Coach 1: Anticipation, Coachability, Adaptability, Positive Attitude), and had possible overlapping characteristics (Coach 1: Resilience- Positive Attitude- On pitch bravery; Coach 2: Decision Making- Adaptability). This process informed the decision on which attributes were removed (Anticipation, Coachability, Adaptability, Positive Attitude, On pitch bravery) or included within the Hull Soccer Behavioural Scoring Tool. Furthermore, it informed the decision of which specific operational definition was selected for each individual attribute.

### Stage three—Establishing face validity

Following analysis from the online surveys data sets, the Hull Soccer Behavioural Scoring Tool was produced with seven behavioural attributes, each behaviour was identified by one operational definition (e.g., Resilience: *Positive attitude after a mistake; how they handle disappointments; ability to overcome adversities; not wanting to give up; remain strong willed; strong work ethic*). This process enhanced content validity as the design was based upon what ‘expert’ practitioners as a whole thought best represented each attribute [29]. In addition, each operator was afforded the opportunity to gain familiarity of using the Hull Soccer Behavioural Scoring Tool. The Hull Soccer Behavioural Scoring Tool was pilot tested which consisted of two coaches (Coach 1: FA UEFA B coaching licence, 5 years professional coaching experience; Coach 2: FA UEFA A coaching licence, 16 years professional coaching experience, sports marketing degree) from a professional soccer academy who used the Hull Soccer Behavioural Scoring Tool within everyday training for three weeks. Adapting previously published methods [26,27], coaches provided detailed critical feedback on the useability and content of the final Hull Soccer Behavioural Scoring Tool with specific reference to the design and language for the operational definitions [40]. The coaches were satisfied with the original behavioural attributes and the subsequent operational definitions. The pilot testing took place three weeks before testing and served as the familiarisation period for coaches. Brewer and Jones [26] and Mars [41] argue this period of pilot testing (including an observation training programme) is necessary to produce high levels of observer agreement for both inter-rater and intra-rater reliability. McKenzie and van der Mars [42] agree stating operational definitions alongside observation-training helps increase reliability, which is tested for in stage four and five in the present study.

### Procedure of testing

Twenty male soccer players (under-16 to under-18) were recruited from a professional, UK soccer academy to participate within the study. Inform consent was waived by the ethics committee, given that the present study’s activity did not require anything additional to the players normal training regime. The players were given different coloured numbered bibs for identification purposes and were randomly categorised into four teams by the primary researcher who had no knowledge of players identity or skill level. The games occurred on four consecutive weeks (including one familiarisation week) during the month of April (2022), using adapted previously published SSG [43] methods. Each player contested 14, ten minute four versus four SSG’s on a 3G pitch (18.3 m x 23 m pitch) using a ‘round-robin’ format [43]. The remaining player on each team would fulfil the role as a substitute should any injuries or dropouts from the study occur. To promote continuous play, throw-ins were taken as ‘kick-ins’, there was no goalkeeper however players were permitted only to score from the attacking half. The ‘round-robin’ game sequence accumulated a total of 100–140 minutes of playing time per player and 70–98 individual player grades provided by the Hull Soccer Behavioural Scoring Tool. Between games, to maintain match readiness, players from the teams not playing performed a standardised technical drill involving between ten and twenty minutes of low-intensity recovery. The SSG’s were recorded using a camera (Panasonic 4K digital camera) which was situated on the half-way line. Moreover, the camera was risen by a tripod (Amazon Basics 50-Inch Lightweight Tripod) to gain a good vantage point and provide clear video footage to watch back. In addition, four cameras were situated in each corner of the pitch to collect better sound quality. The sound from each camera was later amalgamated into the central video with the full pitch view (iMovie, Version 10.1.12). This retrospective video helped establish inter-rater and intra-rater reliability highlighted in stages four and five by coaches within the soccer academy. Coaches were given the Hull Soccer Behavioural Scoring Tool (Table 1) and were required to provide scores for players performance on each of the soccer specific behaviours using a 5-point Likert scale (1 = *Poor*, 2 = *Below average*, 3 = *Average*, 4 = *Above Average* and 5 = *Excellent*). The SSG round-robin format was repeated, using the same players and coaches for three weeks. Given that behavioural performance is multi-dimensional [44], the composite scores from each behaviour (i.e., resilience, competitiveness, confidence etc) were also combined to yield a total score which represents a player’s overall ability in that dimension of performance (e.g., psychological).

**Table 1:** The Hull Soccer Behavioural Scoring Tool.

**Player identification (i.e., name/number/bib colour): ……………………………………………………………** **Match identification (i.e., order number): …………………………………………………………………………** **Fixture details (i.e., age grouped/maturity grouped): ……………………………………………………………**				
*Please provide a score (1 [poor]- 5 [excellent]) for each psycho-social behaviour displayed by the player during his/her* ***whole*** *performance*. *Please ensure you score the players* ***immediately*** *after their performance*. *Key*: *1 [Poor] = never displayed evidence*. *2 [Below Average] = rarely displayed evidence*. *3 [Neutral] = sometimes displayed evidence*. *4 [Above Average] = often displayed evidence*. *5 [Excellent] = always displayed evidence*.				
**Resilience** *Positive attitude after a mistake; how they handle disappointments; ability to overcome adversities; not wanting to give up; remain strong willed; strong work ethic*. *Please insert ‘x’ where you deem appropriate*.				
***1 –[Poor]***	***2 –[Below Average]***	**3 –[Neutral]**	***4 –[Above Average]***	***5 –[Excellent]***
				
***Notes*:**				
**Competitiveness** *Resolve; desire; hunger; strong willed; determination; intense; fighting approach towards winning the ball; winning mentality; hard worker; committed dedicated to the cause; putting their body on the line to block/ stop shots and crosses*. Please insert ‘x’ where you deem appropriate.				
***1 –[Poor]***	***2 –[Below Average]***	**3 –[Neutral]**	***4 –[Above Average]***	***5 –[Excellent]***
				
***Notes*:**				
**Confidence** *Confident within a group; brave; wants to be involved; wants the ball; wants the ball under pressure; confident to be able to get into positions to receive the ball all the time; have the guts to try and fail and do something different; belief in themselves; no fear of failure*. Please insert ‘x’ where you deem appropriate.				
***1 –[Poor]***	***2 –[Below Average]***	**3 –[Neutral]**	***4 –[Above Average]***	***5 –[Excellent]***
				
***Notes*:**				
**Decision Making** *The ability of the performer to select and execute an appropriate action in a given situation; anticipate what is likely to happen prior to the event occurring*. Please insert ‘x’ where you deem appropriate.				
***1 –[Poor]***	***2 –[Below Average]***	**3 –[Neutral]**	***4 –[Above Average]***	***5 –[Excellent]***
				
***Notes*:**				
**Maintaining Composure Under Pressure** *The ability to remain relaxed and handle pressure in different scenarios when performance begins*. Please insert ‘x’ where you deem appropriate.				
***1 –[Poor]***	***2 –[Below Average]***	**3 –[Neutral]**	***4 –[Above Average]***	***5 –[Excellent]***
				
***Notes*:**				
**Communication** *Can have a dialog with players and coaches; talks during the game; ability to listen to both players and coaches; have positive interactions with peers; prepared to ask questions of players and coaches; appropriate body language*. Please insert ‘x’ where you deem appropriate.				
***1 –[Poor]***	***2 –[Below Average]***	**3 –[Neutral]**	***4 –[Above Average]***	***5 –[Excellent]***
				
***Notes*:**				
**X-Factor** *The ability to be creative and produce work that is both novel (i*.*e*, *unexpected*, *original) and appropriate (i*.*e*, *useful)*. Please insert ‘x’ where you deem appropriate.				
***1 –[Poor]***	***2 –[Below Average]***	**3 –[Neutral]**	***4 –[Above Average]***	***5 –[Excellent]***
				
***Notes*:**				

### Stage four—Inter-rater reliability

Two external coaches (Coach 1: FA UEFA B coaching licence, 3 years professional coaching experience; Coach 2: FA UEFA B coaching licence, 20 years professional coaching experience, FA Youth Award, BSc Sports & Exercise Science) scored the same players using retrospective video analysis to assess reliability between practitioners. Video footage was imported in to specialised video analysis software (Catapult, Vision, Catapult Sports, Australia) and the files were synced to permit the coaches to select the most appropriate angle to review the footage. To reduce the possibility of observer drift (the tendency for operators to interpret attributes differently, usually caused through mental fatigue of coding over long time periods; [41]), coaches were encouraged to code the footage for a maximum of an hour, starting and ending at exactly the same time [41]. Prior to formal analysis, the coaches were required to watch two, full SSG videos which served as a familiarisation period for the Hull Soccer Behavioural Scoring Tool. Following this, coach scoring took place during the month of May (2022), with subsequent reliability analysis being conducted eight weeks later. Mars [41] acknowledges two observers is sufficient to achieve interobserver agreement. Given the complexity and multi-dimensional nature of behavioural performance, any agreement above 80% would be deemed as acceptable inter-rater reliability [41], although Lacy and Darst [45] study required observers to achieve at least 85% agreement.

### Stage five—Intra-rater reliability

Finding an acceptable level of intra-rater reliability determines whether the Hull Soccer Behavioural Scoring Tool is accurately consistent between the same operator, watching the same performance, on two separate occasions. To help establish this, and as previously mentioned above (see *Procedure of testing*), the games were recorded to allow the same two coaches use of the Hull Soccer Behavioural Scoring Tool (visualised in Table 1) to retrospectively score players in a game they have already scored live. A number of authors including Mars [41], Darst [46] and Brewer and Jones [26] recommend a minimum of one week between intra- rater coding. However, the present study used four weeks between coding live and retrospective games to further avoid the intra-rater reliability data set being influenced by memory which subsequently increases the strength of intra-rater reliability values [41]. Specifically, retrospective coach scoring took place during the month of May (2022), with subsequent reliability analysis being conducted across the month of June (2022). Again, an acceptable intra-reliability agreement is 80–85% [41].

## Results

### Stage two—Establishing content validity

The aggregated (i.e., sum of *strongly disagree* and *disagree*; sum of *strongly agree* and *agree*) level of practitioner agreement for each of the different operational definitions ‘communication’ (72.7%, 86.4%, 91.0%) and ‘maintaining composure under pressure’ (72.7%, 81.8%, 86.4%) showed little difference between definition. ‘Resilience’ (63.6%, 77.3%, 95.4%, 95.5%) and ‘anticipation’ (68.1% 90.9%, 100.0%) both had clear, preferred definitions which was evidenced by higher levels of practitioner agreement. ‘Decision making’ (50%, 54.6%, 90.9%), ‘competitiveness’ (59.1%, 63.6%, 90.9%), ‘positive attitude’ (40.9%, 40.9%, 77.3%), ‘X-factor’ (40.9%, 54.5%, 72.8%) and ‘coachability’ (59.1%, 63.6%, 90.9%, 90.9%) all had one—two preferred operational statements. ‘Adaptability’ agreement had one lower score at 27.3% whilst the remaining two definitions scored 90.5% and 95.4% respectively. There was no practitioner consensus for ‘match presence’ (36.4%, 45.4%, 63.6%) and on ‘pitch bravery’ (54.6%, 63.7% and 68.2%) operational definitions. A threshold of >70% was considered acceptable for each attribute operational definition [39] (Table 2).

**Table 2:** Summary of highest individual and aggregated (i.e., sum of strongly disagreed and disagreed; sum of strongly agreed and agreed) agreed operational definitions for each attribute. Survey question: *Which definition do you feel best represents ‘…’ in a soccer context*?.

Chosen operational definitions	Strongly disagree	Disagree	Undecided	Agree	Strongly agree	Aggregated disagree	Aggregated agree
***Coachability*:** *Ability to be coached; willing to learn; coachable; good learners; responsive to coaches [9].*	0.0%	9.1%	9.1%	50.0%	31.8%	9.10%	81.8%
***Adaptability*:** *Adapt skills to game situations; how they react to information given to them, or react to how they perceive the game situation unfolding [9].*	0.0%	4.5%	0.0%	54.5%	40.9%	0.0%	95.4%
***Decision making*:** *The ability of the performer to select and execute an appropriate action in a given situation [47].*	0.0%	0.0%	9.1%	36.4%	54.5%	0.0%	90.9%
***Positive attitude*:** *The ability to remain strong willed; work hard; always looking at the positives from performances; always wanting to improve*. *Not allowing outside life to affect the aforementioned qualities*.	0.0%	4.5%	18.2%	36.4%	40.9%	4.5%	77.3%
***Resilience*:** *Positive attitude after a mistake; how they handle disappointments; ability to overcome adversities; not wanting to give up [9,22].*	0.0%	0.0%	4.5%	59.1%	36.4%	0.0%	95.5%
***X-Factor*:** *The ability to be creative and produce work that is both novel (i.e., unexpected, original) and appropriate (i.e., useful) [54].*	0.0%	0.0%	27.3%	45.5%	27.3%	0.0%	72.8%
***Competitiveness*:** *Resolve; desire; hunger; strong willed; determination; intense; fighting approach towards winning the ball; winning mentality [9,22].*	0.0%	0.0%	9.1%	36.4%	54.5%	0.0%	90.9%
***Confidence*:** *Confident within a group; brave; wants to be involved; wants the ball; wants the ball under pressure; confidence to get into positions to receive the ball all the time; have the guts to try and fail and do something different; belief in themselves; no fear of failure [9,22].*	0.0%	0.0%	0.0%	72.7%	27.3%	0.0%	100.0%
***Maintaining composure under pressure*:** *The ability to remain relaxed and handle pressure in different scenarios when performance begins [48].*	0.0%	4.5%	9.1%	59.1%	27.3%	4.5%	86.4%
***Match Presence*:** *The ability to pay attention to what is most important in any situation while ignoring other distractions [49,50].*	0.0%	13.6%	22.7%	40.9%	22.7%	13.6%	63.6%
***Communication*:** *Can have a dialog with players and coaches; talks during the game; ability to listen to both players and coaches; have positive interactions with peers; prepared to ask questions of players and coaches; appropriate body language; can communicate both verbally and non-verbally [9].*	0.0%	4.5%	4.5%	45.5%	45.5%	4.5%	91%
***On pitch bravery*:** *To put their body on the line to block/ stop shots and crosses. This requires no great technique, just being brave and close enough to block the ball [51].*	4.5%	18.2%	9.1%	59.1%	9.1%	22.7%	68.2%
***Anticipation*:** *This is the ability of the performer to predict what is likely to happen prior to the event occurring [47].*	0.0%	0.0%	0.0%	68.2%	31.8%	0.0%	100%

### Stage three—Establishing face validity

Response data showed ‘resilience’, ‘competitiveness’ and ‘decision making’ were all equally valued (90.9%) as more important when compared with other attributes. A small distribution (within 10%) was shown between ‘maintaining composure under pressure’ (72.8%), ‘match presence’ (68.1%), ‘on pitch bravery’ (63.7%), ‘confidence’ (63.6%), ‘positive attitude’ (63.6%) and ‘adaptability’ (63.6%). Following this, a secondary level hierarchy was established, displaying a small distribution between attributes which consisted of ‘anticipation’ (54.6%), ‘coachability’ (54.5%) and ‘communication’ (50.0%). Finally, responses showed ‘X-factor’ was valued significantly less than any other attribute with only 9.1% valuing it as most important. Practitioners’ individual and aggregated (e.g., sum of 1 and 2; 4 and 5) scores for order of importance are presented in Table 3.

**Table 3:** Summary of individual and aggregated (e.g., sum of 1 and 2; 4 and 5) rated order of importance. *Key*: *1 being the least important and 5 being the most important*.

Order of Importance	1	2	3	4	5	Aggregated 1&2	Aggregated 4&5
**Resilience**	9.1%	0.0%	0.0%	31.8%	59.1%	9.1%	90.9%
**Competitiveness**	0.0%	9.1%	0.0%	36.4%	54.5%	9.1%	90.9%
***Decision Making***	4.5%	4.5%	0.0%	36.4%	54.5%	9.0%	90.9%
***Maintaining Composure Under Pressure***	0.0%	9.1%	18.2%	36.4%	36.4%	9.1%	72.8%
***Match Presence***	4.5%	9.1%	18.2%	54.5%	13.6%	13.6%	68.1%
***On Pitch Bravery***	9.1%	9.1%	18.2%	45.5%	18.2%	18.2%	63.7%
***Confidence***	0.0%	9.1%	27.3%	54.5%	9.1%	9.1%	63.6%
***Positive Attitude***	0.0%	18.2%	18.2%	40.9%	22.7%	18.2%	63.6%
***Adaptability***	4.5%	18.2%	13.6%	31.8%	31.8%	22.7%	63.6%
***Anticipation***	4.5%	9.1%	31.8%	36.4%	18.2%	13.6%	54.6%
***Coachability***	4.5%	0.0%	40.9%	27.3%	27.3%	4.5%	54.5%
***Communication***	4.5%	13.6%	31.8%	40.9%	9.1%	18.1%	50.0%
***X-Factor***	4.5%	22.7%	63.6%	9.1%	0.0%	27.2%	9.1%

### Stage four—Inter-rater reliability

**Fig 2:**
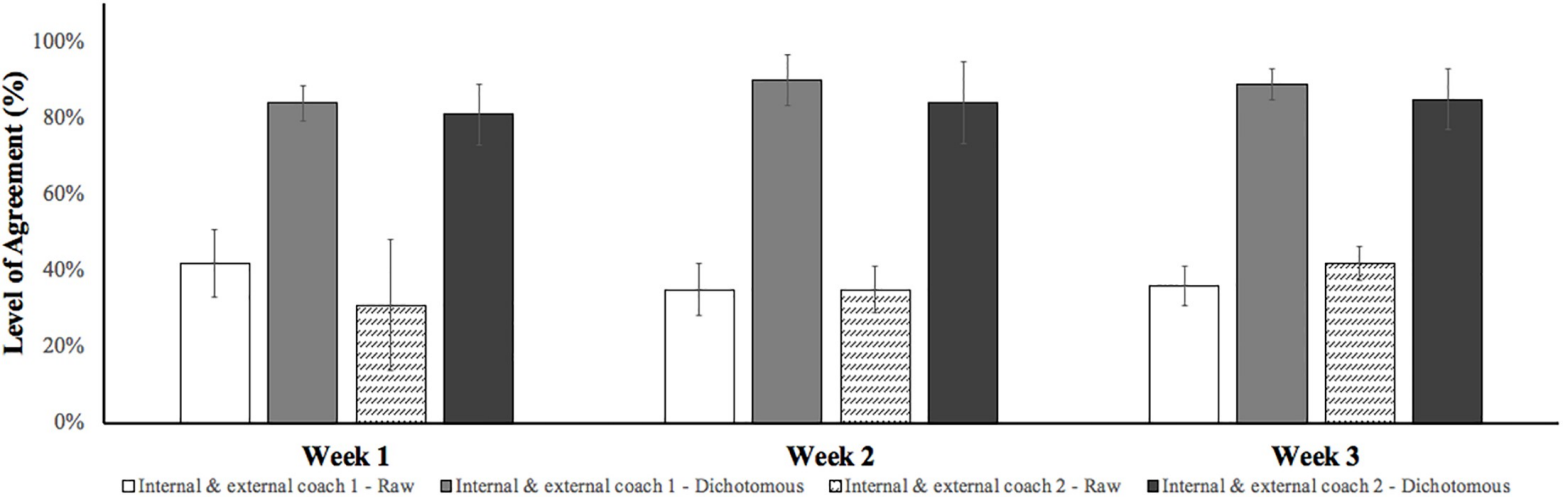
Mean percentage agreement (± S. D) for inter-rater (between two operators) reliability.

Once the external coaches had coded all the games using video analysis for all subsequent weeks, an inter-rater reliability coefficient, using the sum of agreements/ (agreements + disagreements) was established [41]. Currently, there’s no gold standard for level of agreement when assessing reliability for behavioural scoring tools, however Mars [41] deems 80–85% as being sufficiently high. Original results for internal coach (live player assessment) and external coach 1 (retrospective video player assessment) revealed mean agreement scores of 42.0% (± 8.9%), 35.1% (± 6.9%) and 35.7% (± 5.0%) across three subsequent testing weeks. Original results for internal coach (live player assessment) and external coach 2 (retrospective video player assessment) recorded mean agreement scores of 31.3% (± 1.7%), 35.1% (± 6.1%) and 42.0% (± 4.5%). Although these results don’t reach an acceptable level of agreement, this study used Peabody’s [52] version of the Likert scale using a dichotomous approach whereby scores were calculated using response intensity around the mid-point (e.g., anything within 1 score of each other were accepted [4: Above Average; 5: Excellent]). This method substantially altered results and the differences between the raw data and the dichotomous scale can be viewed in Fig 2. Using the dichotomous scales, results between internal coach and external coach 1 (live player assessment) revealed mean scores of 83.9% (± 4.6%), 89.9% (± 6.6) and 89.3% (± 4.1%) across the three subsequent testing weeks. Again, using dichotomous scales, internal coach (retrospective live player assessment) and external (retrospective video player assessment) coach 2 revealed slightly lower mean reliability scores of 81.3% (± 7.9%), 83.9% (± 10.8%) and 84.8% (± 7.9%). Whilst some of the coefficients fell below Mars’ [41] 80–85% acceptable level of agreement, all dichotomous mean scores surpassed the minimum accepted 80% level and therefore were deemed as successful for inter-rater reliability testing.

### Stage five—Intra-rater reliability

**Fig 3:**
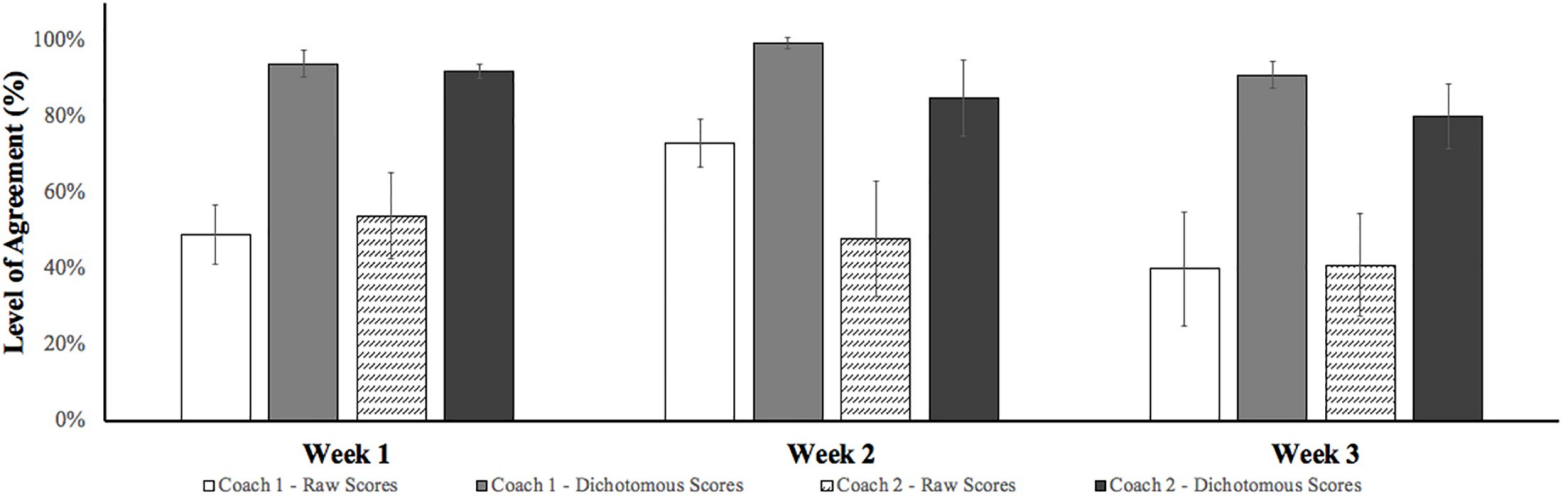
Agreement level mean values (± S. D) for intra-rater (between individual operators) reliability.

Once internal coaches (live player assessment) had re-coded the games for each of the 3 separate weeks, the intra-rater reliability (i.e., live player assessment scores versus retrospective video player assessment) coefficient was calculated using the same sum of agreements/ (agreements + disagreements) [41]. Again, whilst there’s no gold standard for level of agreement, Mars [41] deems 80–85% to be sufficiently high. Original results for internal coach 1 revealed relatively similar mean scores across week 1 and week 3 scoring 49.1% (± 7.9%) and 40.2% (± 15.0%) respectively, whilst week 2 was considerably higher scoring 73.2% (± 6.3%). Internal coach 2 revealed mean scores of 53.6% (± 11.3%), 47.6% (± 15.3) and 41.1% (± 13.5%). Although these results did not achieve the accepted agreement level, as previously mentioned the present study used Peabody’s [52] version of the Likert scale by using a dichotomous approach. Using this scale, the intra-rater reliability achieved substantially higher levels of agreement with internal coach 1 mean scores ranging from 93.7% (± 3.4%), 99.4% (± 1.5%) and 91.1% (± 3.6%) across the three testing weeks. Internal coach 2’s mean scores were also higher at 92.0% (± 1.8%), 85.1% (± 1.0%) and 80.4% (± 8.5%) across three subsequent testing weeks. Again, whilst some of the coefficients did not satisfy Mars’ [41] acceptable agreement level, overall, all mean dichotomous scores surpassed a level of agreement needed to be deemed as an excellent score for intra-rater reliability testing (Fig 3).

## Discussion

Currently, whilst psycho-social behaviours are highly valued by practitioners [8], there is no match-play behavioural scoring tool to measure these attributes. Therefore, this study aimed to develop a valid and reliable tool (i.e., Hull Soccer Behavioural Scoring Tool) for coaches to assess desirable player behaviours during match-play performance. In addition, the study investigated which of the different psycho-social attributes professional soccer practitioners valued as least and most important for talent development. The main findings of this study were three-fold: 1) Practitioners rated player resilience, competitiveness and decision making as the most important psycho-social attributes (Table 3). There was a relatively even distribution between the remaining attributes with the exception of X-factor which was rated considerably lower (Table 3); 2) With the exception of two attributes where the level of agreement was deemed unacceptable and was therefore removed, practitioners agreed on one operational definition to best represent the specific behaviour in question when presented with three to five different options (Table 2); 3) Both inter-rater (Fig 2) and intra-rater (Fig 3) reliability scores satisfied the 80–85% agreement threshold cited by Mars [41] when implementing a dichotomous approach [52].

Using professional soccer practitioner survey responses, this study established a hierarchy of psycho-social attributes that are considered desirable within youth soccer and occur in match-play performance. Results here indicate that player resilience, competitiveness and decision making were all perceived as the most important psychological behaviours. Whilst X-factor was considered the least important of all attributes. These findings are comparable to previous studies [8,9,30] which have comprehensively assessed professional soccer practitioners’ perceptions of psycho-social attributes for talent identification purposes. However, unlike Larkin and O’Connor [9] the hierarchy of perceived importance within the present study yielded different results. For instance, coachability and positive attitude were rated as most important by Larkin and O’Connor [9], whereas 54.5% and 63.6% of respondents within the present study rated these attributes as most important respectively. In line with Larkin and O’Connor’s [9] findings, decision making was rated as most important by 90.9% of practitioners in the present study. In addition, anticipation and confidence were rated moderately important (or middle of the hierarchy) by practitioners which mirrors the findings reported by Larkin and O’Connor [9] and which suggests that despite the current study’s limited sample, findings here corroborate a number of the psycho-social attributes identified by researchers [35] and practitioners as being most important for the talent transition process during youth soccer [8,9]. That said, whilst Larkin and O’Connor [9] found that X-factor was rated as moderately important, only 9.1% of practitioners in the current study valued X-factor as most important. This might be considered surprising since research has highlighted creativity (which is inextricably linked with X-factor) is an important factor for player selection in youth soccer [53] and is directly linked to game performance and progressing into later rounds of senior tournament competitions [54]. Despite soccer player creativity being perceived vital for creating chances, scoring goals and a key principle of play [55], it is mainly associated with attacking players [56,57]. Given that the present study’s objective was to create a general soccer specific tool which can be used across playing positions, the perceived importance assigned by the surveyed practitioners may be influenced by playing position bias and/or philosophy. This may have influenced the present study’s survey response data as many practitioners might downplay the importance of X-factor if they perceive this attribute to be ‘more useful’ in some playing positions but less in others [56]. Despite some youth soccer coaches stating that players ability to show creativity in one-versus-one scenarios was a defining key performance indicator during talent identification, others may believe that defending isn’t a priority until later in the youth development phase (under-12 to under-16) and therefore focus their training on in-possession techniques [9], which could suggest X-factor is valued by youth team practitioners regardless of playing position. In addition, creativity is closely linked to other psycho-social attributes and so respondents in the present study may have rated alternative attributes more highly with the assumption creativity may naturally arise. To illustrate, coaches mention players must have a good level of confidence in order to be creative as they try new and unpredictable skills within a constantly changing situational context [9]. Moreover, creativity requires players to take risks which will inevitably lead to occasional mistakes or errors, as a result this can potentially make players resilient to bad performances if they are reassured by coaches that mistakes are an important part of the learning process [58,59]. There is also considerable evidence to suggest that player creativity is strongly related to the decision making process because players have to make quick and decisive choices to solve game-related challenges within the time constraints associated with soccer performance [60,61]. Interestingly, in the present study decision making was rated joint most important and this could suggest the respondents selected decision making as most important without fully understanding the skills which make up the attribute X-factor, especially given that literature highlights an overlap between the two qualities [60,61].

Resilience, competitiveness and decision making were all jointly rated as the most important behavioural attributes in the present study. We postulate resilience was rated highly because of a range of characteristics that combine to make up this attribute [62]. To illustrate, while resilience can be defined as having the capacity to bounce back from adversities [30], the attribute is seen as a significant component of mental toughness [62]. Mental toughness is thought to facilitate the development of various coping strategies that help players overcome obstacles [33] thereby increasing their confidence to deal with setbacks [30,33] and reducing the fear of failure [63]. This is important within youth soccer for a number of reasons. Firstly, problem-focused coping strategies are associated with higher levels of resilience [63] which may allow players to play with a level of freedom and confidence that makes them stand out especially as they transition through youth development into a first team environment [30,33]. By contrast, the use of avoidance-focused coping strategies, for example trying to block out and ignore uncomfortable situations or events, means that athletes do not learn how to develop resilience to adversity. Problem-focused coping strategies are important because they equip players with the ability to control their thoughts and emotions to remain positive after mistakes [35]. The use of such strategies may encourage athletes to persevere and trust their own ability, and this increases their resilience to challenges and setbacks they inevitably face along the developmental pathway [30,33]. Therefore, inclusion of psychological behaviours such as resilience within the newly proposed Hull Soccer Behavioural Scoring Tool increases ecological validity.

The content validity of the Hull Soccer Behavioural Scoring Tool was established using two recommended methods which included an initial literature review [27,64], followed by a panel of professional soccer practitioners rating the shortlisted psychological behaviours using on an online survey with a 5-point Likert scale [65]. This process was conducted to ensure the cross-sectional survey fully covered all psycho-social attributes with different variables whilst also establishing the said behaviours have relevance, utility and representativeness within soccer match-play [26,28,29,65]. The first stage of establishing content validity was to survey soccer practitioners to provide a rating to reflect how well each of the stated operational definitions represent the psychological behaviour. Whilst this helped provide a first draft of the instrument, it was considered equally important to ensure that the proposed new Hull Soccer Behavioural Scoring Tool possessed the capacity to measure what it was designed to measure and has practical utility within a real-world environment [27]. To enhance face validity, two professional soccer academy coaches were requested to use the Hull Soccer Behavioural Scoring Tool during daily practices and provide detailed feedback on if they thought each behavioural definition captured the players characteristics within a real-world, applied soccer context, supported by guidance on whether the operational definitions were suitable or required further adaptation [27]. Both academy soccer coaches were in agreement that the design, listed behaviours and subsequent operational definitions were suitable for use and required no further alteration consequently showing the proposed Hull Soccer Behavioural Scoring Tool possessed suitable content validity.

Having finalised the content validity of the Hull Soccer Behavioural Scoring Tool, the inter-rater and intra-rater reliability was assessed. In the present study, analysis of raw data sets revealed low levels of agreement between both inter-rater and intra-rater assessments with scores ranging between 31.3%—42.0% and 40.2%—73.2% respectively. These findings contrast similar previous studies assessing soccer coach behaviours, which demonstrated enhanced levels of reliability (inter-rater: 76%—86%, intra-rater: 75%—88%; [27]) and which failed to surpass acceptable thresholds (80–85%) set by Mars [41] due to the complexity and number of behaviours being assessed. Although the raw results for inter-rater and intra-rater reliability did not achieve the accepted agreement level, as previously mentioned the present study used Peabody‘s [52] version of the Likert scale by devising a dichotomous approach. Although a 5-point Likert scale isn’t sensitive enough to detect very small change [66], Lissitz and Green [67] suggest, depending on the research’s objectives, having a scale larger than 5 wouldn’t usually be useful because the variability within reliability scores tend to plateau at 5 scale points. In response to what seemed to be low raw reliability scores, a dichotomous approach was used whereby level of agreement was established through the intensity of the mid-point [52]. For example, if *1*: *poor* and *2*: *below* average were selected this was deemed as an accepted level of agreement, however if *1*: *poor* and anything above *3*: *neutral* were selected this was deemed as disagreement. We used this scale because when users provide a score, only 10% of response intensity contribute to total score variation for the extent of agreements or disagreements. Whereas 70–80% contributed to direction (e.g., agree or disagree) [52,66]. Using this scale, the inter-rater and intra-rater reliability data sets substantially increased level of agreement with scores ranging between 81.3%—89.9% and 88.4%—99.4% respectively. Whilst some of the coefficients did fall below Mars’ [41] acceptable agreement level, overall, all mean dichotomous scores surpassed the necessary level of agreement needed to be deemed as successful for inter-rater and intra-rater reliability testing.

Whilst the results from this study suggest that the Hull Soccer Behavioural Scoring Tool offers a valid and reliable tool for the identification of youth soccer player behavioural characteristics, there are a number limitations that require consideration. First, reliability data were obtained during a four versus four game format with specific pitch sizes. Both pitch size and number of players can alter the number of decisions [68], competitive actions (e.g., defensive duels) [69], space for creativity and dribbling skills [70], and player resilience by having more or less opportunity for failure [30,71]. Therefore, such contextual factors may influence behavioural performance during different game conditions, especially during 11 versus 11 competitive fixtures–a format beyond the scope of the present study. That said, small-sided games are commonly used within soccer clubs [72,73] for technical and physical conditioning [74,75], and also employed for talent identification purposes [76]. In addition, given all participating coaches work in professional youth soccer and hold professional coaching qualifications (e.g., UEFA B or UEFA A), there is a possibility of a systematic bias among these coaches through experience and education. Future research should consider using a larger sample size with inexperienced and experienced operators (see Brewer and Jones [26]). Finally, given there seems to be no uniformly accepted framework backed by theoretical knowledge to help guide practitioners current work [77,78], we cannot definitively state that the Hull Soccer Behavioural Scoring Tool is the best instrument for assessing desirable behaviours, however our results indicate it offers considerable promise as a tool to help practitioners with the ongoing (de)selection process.

To conclude, this study followed a rigorous method to successfully attain its aims of developing a valid and reliable match-play Hull Soccer Behavioural Scoring Tool for youth soccer players competing in small-sided games. Validity was established using numerous methods including, an extensive literature review, an online survey with professional practitioners and a pilot test using a draft Hull Soccer Behavioural Scoring Tool during small-sided games. Reliability was established using both inter-rater and intra-rater reliability tests to an acceptable >80% level of agreement.

The Hull Soccer Behavioural Scoring Tool has been designed as a means to objectively measure behavioural characteristics in youth soccer players. This is important given the present issues surrounding relative age in childhood [79,80] and maturation in early adolescence [79,80] can influence a players physical capabilities [18,80] and potentially confound a practitioners view on ‘talent’ [81]. The practical applications are for constituent clubs to use the Hull Soccer Behavioural Scoring Tool as a method to assist with holistically assessing talent for the ongoing (de)selection process. Importantly, the Hull Soccer Behavioural Scoring Tool is not designed to devalue players, but to support their psycho-social development and assist practitioners in the talent development process. Future research should consider further testing to strengthen the validity of the Hull Soccer Behavioural Scoring Tool and confirm its utility across more settings (i.e., different age categories; 11 versus 11 game format etc).

### Hull Soccer Behavioural Scoring Tool (Table 1) Online Access

https://docs.google.com/document/d/e/2PACX-1vRyQGNSNPmnob7FN1P2KdNWZIHCZsZm5rmtU2XyPNjmHmr1eaqZkOSV8_RxHopJXA/pub

## Supporting information

S1 File (XLSX)
